# Educational training programs on intimate partner violence in pregnancy for midwives/student midwives: A scoping review

**DOI:** 10.18332/ejm/189282

**Published:** 2024-07-05

**Authors:** Emmanouela Manoli, Christiana Kouta, Maria Karanikola, Nicos Middleton, Eleni Hadjigeorgiou

**Affiliations:** 1 Department of Nursing, School of Health Sciences, Cyprus University of Technology, Limassol, Cyprus

**Keywords:** evaluation, educational training, midwives, student midwife, intimate partner violence, pregnancy

## Abstract

**INTRODUCTION:**

Educational strategies for preventive screening and effective interventions in midwives are needed to improve clinical practice and outcomes for abused women and their families. This scoping review aimed to describe available educational training programs on intimate partner violence (IPV) in pregnancy for midwives/student midwives.

**METHODS:**

A scoping review of the literature, which was published in English from January 2010 to March 2023, in PUBMED, EBSCO, and CINAHAL databases, was applied. The following keywords were used in the search: ‘evaluation’, ‘educational training’, ‘course’, ‘midwives’, ‘student midwife’, ‘intimate partner violence’, ‘pregnancy’, combined with AND and OR Boolean operators. The included studies focused on training programs/courses for midwives/student midwives regarding intimate partner violence.

**RESULTS:**

A total of 9 studies were eligible for inclusion, describing six programs for midwives and 3 for student midwives. Educational interventions varied in length (e.g. a few hours to weeks) and educational approaches such as multidisciplinary sessions, lectures, theory, role-playing, practice in screening, group activities, watching videos, and case reports discussion. The programs had similar content, including raising awareness of violence, defining it, discussing gender roles, the impact of IPV on women’s health, referral agencies, and the laws regarding violence in each country.

**CONCLUSIONS:**

This scoping review highlighted a lack of educational programs on intimate partner violence during pregnancy, suggesting that new programs need to be developed based on contemporary clinical practices and recommendations for midwifery education.

## INTRODUCTION

Intimate partner violence (IPV) is a major public health problem that affects one-third of women worldwide^1^. WHO reported that pregnant women are more vulnerable to IPV due to changes in their physical, emotional, social, and economic situation, and defines IPV as ‘the behavior by an intimate partner or ex-partner that causes physical, sexual or psychological harm, including physical aggression, sexual coercion, psychological abuse, and controlling behaviors’^1^.

Meta-analysis of risk factors on violence during pregnancy showed that predictors of abuse during pregnancy included abuse before pregnancy, lower education level, pregnancy being unintended by either the victim or the perpetrator, lower socioeconomic status, and being unmarried^2^. Immigrant women are more vulnerable to IPV due to their economic insecurity, language barriers, family separation, social isolation, and discrimination^3^. IPV during pregnancy does not only affect women’s reproductive health but also imposes fatal and non-fatal adverse health outcomes on the growing fetus due to the direct trauma of abuse to a pregnant woman’s body^4^. IPV prevalence is higher than many common obstetric conditions^5^, such as first/second trimester bleeding, late entry into antenatal care^6^, and preterm labor^7^; moreover, infants of women reporting IPV in pregnancy are more likely to experience low birth weight^8^, under-nutrition, and higher rates of mortality^9^. Additionally, women affected by IPV are less likely to breastfeed their babies^5^ and more likely to experience mother-to-infant bonding failure at one month postnatal^10^.

Midwives have a central role in the provision of maternity care and are usually the first point of contact with pregnant women; thus, they are expected to routinely and accurately identify and support survivors of IPV^11^. Developing an empathic relationship with women may allow them to feel safe and confident to discuss sensitive matters like violence in their relationship and further be assessed for IPV^12^. However, there are a number of barriers for midwives to openly communicate with women who have experienced violence, such as inadequate education and training on the topic and, subsequently, a lack of relevant skills and competencies^13^. As a result, midwives usually describe reluctance and lack of confidence to discuss IPV-related issues with women^14^. Nevertheless, addressing experiences of violence in those cared for can be challenging for midwives; adverse emotional responses in midwives, such as shame, criticism, and uncertainty, along with a lack of knowledge and relevant skills, may lead to ignorance of women’s experiences of IPV, and subsequently exclusion of this topic from their care plan^15^.

Literature suggests that effective educational strategies for midwives are needed to improve their clinical practice and outcomes for abused women and their families^11-16^.

In education, the inclusion of screening by maternity health professionals has been debated not only in terms of identifying asymptomatic patients but also with a focus on a combination of identifying such patients and subsequently intervening to influence desired outcomes. This intervention may lead to reduced future violence, enhanced quality of life, improved pregnancy outcomes, or similar positive effects^16^. WHO^1^ suggested that healthcare providers need to be prepared to provide immediate support and referral to women experiencing IPV. It also recommends IPV training that incorporates safety planning, communication, and referral to specialist agencies and addresses staff attitudes toward victims of IPV. Additionally, the International Confederation of Midwives^17^ emphasizes the need to strengthen the quality of midwifery education and essential competencies for midwifery practice in its recommendations. These recommendations guide the training of midwives, focusing on crucial aspects. This includes safeguarding privacy and confidentiality, offering information to all women about available sources of help regardless of whether there is disclosure about violence, and routinely inquiring about safety at home and at work. Furthermore, they emphasize recognizing potential signs of abuse from physical appearance and emotional affect, identifying related risk behaviors such as substance abuse, and providing special support for adolescents and victims of gender-based violence, including rape. The recommendations also stress facilitating referrals to community resources while assisting in locating a safe setting as needed.

Training programs need to address common myths associated with violence to prompt positive attitudes, focus on knowledge and preparation for routine inquiry, provide information on local resources, and promote adherence to best practices^11^. Training programs should not be just for health professionals in the hospitals but also for the student midwives and nurses who have increased knowledge and awareness of violence against women because they will interact with women when they start working in the profession^18^. Considering that universities’ midwifery programs include curriculum content training in the prevention, detection, and support of victims of IPV^19^ so that students can act and speak up when there are violations of human rights^1^.

The aim of this scoping review was to describe available educational training programs for midwives/student midwives regarding intimate partner violence in pregnancy, with a focus on: 1) their features and 2) areas of effectiveness.

## METHOD

### Study design

A scoping review of the literature took place according to the following steps: 1) report of the objectives of the review, 2) systematic search of scientific data, according to predefined criteria, to collect the sample studies of the study, 3) assessment of the methodological quality of the reviewed studies to be included in the sample, and 4) critical and systematic report of the features and main results of the sample studies. The methodology of the present review was reported according to the Preferred Reporting Items for Systematic Reviews and Meta-Analyses extension for Scoping Reviews (PRISMA-ScR) (Supplementary file, and reference therein).

### Search strategy

Αn advanced search in the following databases was applied: PUBMED, EBSCO, CINAHL, using the following keywords alone and in combination, in line with Medical Subject Headings: ‘evaluation’, ‘educational training’, ‘course’, ‘midwives’, ‘student midwife’, ‘intimate partner violence’, and ‘pregnancy’ and combined with AND and OR Boolean operators. The search strategy was performed in January 2023. A rerun of the search was performed in March of 2023. This search was conducted by two members of the research team (EM, EH).

### Inclusion and exclusion criteria

To be considered for inclusion, studies were required to: 1) employ a quantitative research design or to encompass such a design within their methodology (mixed-methods studies), 2) include in the sample midwives and/or student midwives, 3) focus on the effectiveness of a training program or a course, addressing intimate partner violence, 4) be written in English, and 5) be published between January 2010 and March 2023 in a peer-reviewed journal. Conference presentations and articles describing an educational program with no reference to empirically produced data on its effectiveness were excluded.

Two researchers (EM, EH) independently screened the titles and the abstracts of all retrieved articles for eligibility and resolved disagreements by consensus. An extraction sheet to support the data collection procedure and documentation of the reasons for excluding a study was used.

Two members of the research team (EM, EH) assessed the quality of the studies independently, and any disagreements were resolved by consensus. No studies were excluded due to low quality. The researchers evaluated the quality of the reviewed studies using the National Institutes of Health Quality Assessment tool for cross-sectional studies, the Mixed-Methods Appraisal Tool for mixed-methods studies (MMAT), and the Joanna Briggs Institute Critical Appraisal Checklist for Quasi-Experimental Studies (Supplementary file Tables 1–3, and references therein).

Furthermore, each study of the sample was independently reviewed by the two researchers (EM, EH) corresponding to the measures employed in the aims of the review, i.e. target population/sample (midwives, healthcare professionals), duration of intervention, educational method and content and key findings. An extraction sheet specially designed to support this process was used for the data collection procedure, and the reason for excluding a study was documented.

### Data analysis

Data were analyzed in three steps. The first step involved the identification of studies that fulfilled the inclusion criteria, the description of their main methodological characteristics, the development of relevant tables, and the assessment of their methodological quality. The final stage encompassed the organization of the results of the sample studies in relation to the present research objectives and the interpretation of data regarding the effectiveness and features of IPV educational programs. Special focus was placed on the barriers regarding the implementation of such programs.

## RESULTS

All of the studies were published between 1997 and 2015, and study samples included 4274 nurses working from 3 to 25 years either in the community, mental health, residential care facilities, or hospital units.

**Figure 1:**
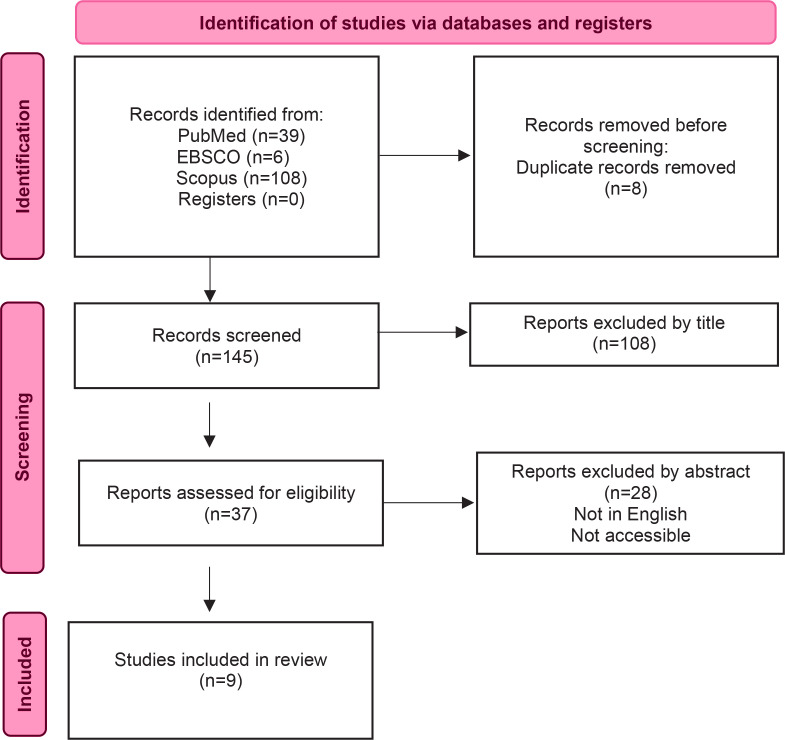
PRISMA flowchart summary of search strategy and study selection

The selection strategy of the sample studies was based on the PRISMA guidelines (Figure 1). The search produced 153 articles. Eight articles were removed as duplicates. One hundred forty-five full-text articles were assessed on relevance. By studying the titles, 108 articles were excluded as they were irrelevant to inclusion criteria objectives. After studying the abstracts of the remaining articles, 28 of them were excluded because they were inaccessible. Only nine articles fulfilled the criterion of assessing the effectiveness of training programs for midwives on IPV. All these nine articles were evaluated and found to be of satisfactory methodological quality according to standardized tools (Supplementary file Tables 1–3).

### Study characteristics

More specifically, the characteristics of the nine studies are shown in Table 1. Three studies were pre-post interventions studies, one was a cross-sectional study, three were experimental studies and two were mixed-methods studies. The sample size of health professionals who participated in these studies ranged from 21 to 408 people, including midwives (n=706) and student midwives (n=389). Studies were conducted in Australia (n=3), Europe (n=3), and Asia (n=3). Three studies included midwives and other health professionals, such as obstetricians and nurses^12,19,20^. A total of six educational programs for midwives and three for student midwives were included in the sample studies. Most of the studies (n=6) identified education strategies for professional midwives who worked in hospital maternity services. One study included community-based midwives while two included educational courses integrated into the midwifery curriculum^18,22^.

**Table 1:** Characteristics of studies evaluating educational training programs on intimate partner violence in pregnancy for midwives/student midwives from January 2010 to March 2023

*Study Year Country*	*Aim*	*Type of study population*	*Duration of intervention*	*Educational method and content*	*Key findings*
Torres-Vitolas et al.^12^ 2010 United Kingdom	To identify maternity and sexual healthcare professionals’ training needs regarding routine inquiry for domestic abuse (DA)	Cross-sectional study Pre-training questionnaires 222 attendees Maternity and sexual health professionals (including doctors, midwives, and nurses)	One day domestic abuse (DA) training session	Educational method: multidisciplinary sessions Content: Domestic abuse awareness, practical training for routine inquiry for domestic abuse, documentation, and referral to the advocacy service.	39.9% of the participants reported limited domestic abuse training. In total, 43.3% of the participants reported that they had encountered at least one disclosure of DA in the 12 months before the training session. 46% of maternity health professionals reported they suspected that a patient was a victim of DA compared to 30.6% of other medical professional staff.
Baird et al.^24^ 2013 United Kingdom	To evaluate the degree to which practice changes identified from a previous evaluation of the Bristol Pregnancy Domestic Violence Programme (BPDVP) for routine inquiry for domestic abuse has been maintained	Follow-up study Mixed-methods -semi-structured questionnaires -focus group interviews Five years after the introduction of the training program 58 midwives	One day program	Educational method: Content: -awareness-raising -policy development -referral pathways and documentation, -particular emphasis on women’s safety.	In this study, 61% of the midwives reported their levels of confidence following training on DA increased, compared to 36% in the first study. Generally, the effect of training on knowledge was significantly increased on the screen of asking and responding to domestic violence.
Jayatilleke et al.^21^ 2015 Sri Lanka	To evaluate the training program’s efficacy in improving public health midwives’ identification and management of IPV sufferers in Kandy	Pre-post intervention study -pre-post intervention survey at 6 months posttraining 408 public health midwives (PHM)	Four days of training	Educational method: Using role-playing and case reports, the trainers discussed how to manage IPV sufferers in different situations and improved the PHMs’ practical IPV skills Content: -gender roles -the types, acts, and health effects of IPV -the domestic violence -prevention law in Sri Lanka -the available supportive services for IPV sufferers in the country -how to identify and assist IPV sufferers	The training program improved practices significantly. 98.5% of midwives identified at least one intimate partner violence suffered compared to pre-intervention rates. All post-intervention scores were at least 50% higher than pre-interview scores (e.g. knowledge, perceived responsibility, self-confidence).
Baird et al.^11^ 2018 Australia	To evaluate the longitudinal impact of a domestic violence training and support program to promote midwives’ routine antenatal inquiry for domestic violence using a mixedmethods design	Mixed-methods design-a survey of midwives at 6 months post-training, -interviews with key stakeholders at 12 months -chart audit data of screening, risk, and disclosure rates (for 16 months). 83 midwives	Seven-hour workshop	Educational method: Included a variety of teaching approaches such as group activities, role-play, and analysis of case studies. Content: -survivor experiences DV during pregnancy -responses by health professionals. -information sharing session from a local domestic and family violence community support and referral agency. -discussion of strategies to overcome potential barriers to routine inquiry in practice. -an opportunity for participants to share concerns and debrief	This study identifies that all follow-up scores were significantly higher than baseline scores. The score for level of preparedness increased by 20%, and knowledge increased by 17%. More than 90% of participants reported improved confidence to undertake the routine inquiry. The chart audit showed that 90% of the women were screened. Only 2% of the women screened disclosed domestic violence, and most women at risk refused the referral.
Baird et al.^25^ 2018 Australia	To evaluate the impact of training on the knowledge and preparedness of midwives and nurses to conduct a routine inquiry about domestic violence (DV) with women during the perinatal period	Pre-post intervention study -pre-post questionnaire 149 Midwives	One day workshop	Educational method: Teaching approaches included lectures, group activities, videos, role-play, and analysis of case studies. Group work sessions encouraged discussion around topics such as what constitutes violence against women and why women may choose to stay with a violent partner. Content: -What constitutes violence against women and why women may choose to stay with a violent partner. -Stereotypical attitudes and myths about DV. -Common misconceptions about DV. -Effects of DV on children and legislation around mandatory reporting for child safety. -The role of midwives and nurses in identifying and supporting women experiencing DV included discussions on responding, boundaries, safety, and record keeping. -Survivor shared her story and experiences of maternity care during pregnancy. -DV community support agency on referral processes and services. A recorded role-play was used to identify respectful communication skills. -Discussion of strategies to overcome potential barriers to routine inquiry.	All post-intervention scores were significantly higher than pre-interview scores (knowledge, preparedness). The score for level of knowledge increased by 19%, and preparedness increased by 31%. 93% of midwives reported the training program improved their awareness of domestic violence and improved their skills for screening and how to respond to a woman’s disclosure of domestic violence.
Smith et al.^23^ 2018 Australia	To increase midwifery students’ confidence in screening for and responding to disclosure of domestic violence in maternity service	Mix-Methods study (quantitative and qualitative questions) 174 student midwives	One day workshop	Educational method: Theory and practice in regard to screening for and responding to domestic violence in pregnancy. Content: Overview of domestic violence in Australia. Discussion of the role of the social worker in assistingmidwives working with women experiencing violence which was presented by a perinatal social worker. Introduction to the ‘Counting Dead Women’ project (Counting Dead Women Australia and Destroy the Joint,2016) presented by an activist and journalist who established Destroy The Joint. Destroy the Joint is an online community that stands for gender equality and civil discourse in Australia. Review of the evidence about the impact of domestic violence in pregnancy and parenting. Responding to the ‘why doesn’t she just leave’ question through the use of the TEDTalk ‘Why Domestic Violence Victims Don’t Leave’ (Morgan-Steiner, 2012). Routine screening for and responding safely to the disclosure of domestic violence in pregnancy through the use of a purposely developed authentic practice video Overview of child protection issues. Self-care strategies for students and midwives.	Midwifery students increased their confidence by responding appropriately to disclosure and assisting women with access to support. The largest increase (47%) in students’ confidence levels was the score for the topic area ‘Providing an appropriate response if the woman discloses current domestic violence’.
Yilmaz^22^ 2018 Turkey	To evaluate the effectiveness of a gender equality course in changing undergraduate midwifery students’ attitudes toward domestic violence and gender roles	One-group before-after quasi-experimental design ‘The Attitudes Towards Domestic Violence Scale’ and ‘The Gender Roles Attitudes Scale’ First-year undergraduate midwifery students (n=64)	Course: 10 sessions for 2 hours	Educational method: Used case reports, visual presentations, and discussions. Content: The gender equality course consisted of two parts. The first part, which related to gender roles, was structured based on the related literature (World Health Organization, 2006; Prime Ministry Directorate General on the Status of Women, 2008b; World Health Organization, 2009; Ecevit et al. 2011; Dökmen 2016). The second part related to DV was structured based on the related literature also (Berman, Barlow, Koziol-McLain, 2005; Jayatilleke et al. 2015; Crombie, Hooker, Reisenhofer 2016).	The course helped the students to develop more positive attitudes toward domestic violence and gender roles. The scores for the attitudes towards domestic violence only increased by a few percent, while the scores for attitudes to gender roles increased by 7%.
Sis Çelik and Aydın^18^ 2019 Turkey	To determine the effect of a course on violence against women on the attitudes of student midwives and nurses towards violence against women and their abilities to recognize the signs of violence	Pretest-post test quasi-experimental design with experimental and control groups. Questionnaire N=78 student midwives and nurses’ experimental group (enrolled in the course) N=73 student midwives and nurses control group (did not choose to enroll in the course)	Course on violence against women: 2-hour lecture every week for 14 weeks, for a t otal of 28 hours	Educational method: Presentations Content: Social and gender equality Definition, types, causes, and symptoms of violence impact of violence on women’s health Legal aspects of violence against women Services offered to women victims of violence Responsibilities of healthcare professionals in the elimination of violence Responsibilities of healthcare professionals and a presentation of healthcare services for victims Violence screening tools Risk assessment, development of security plans, and notification forms.	The results indicated that the difference between pretest and post-test scores averaged across three scales(attitudes towards violence (25%), attitudes of healthcare personnel towards occupational roles in addressing violence against women (29%), and the ability of nurses and midwives to recognize the signs of violence against women (41%) was statistically significant for students in the experimental group and statistically insignificant for students in the control group.
Duchesne et al.^20^ 2020 France	To assess the impact of a brief training for obstetricians and midwives about screening for domestic violence during pregnancy follow-up and to identify barriers to a routine inquiry	Quasi-experimental study -Patients’ survey 13 obstetricians and 8 midwives – attended the intervention 495 patients in the control group 395 experimental group	One hour-anda-half training session	Educational method: Presentations were performed using PowerPoint software. Presenters gave further details, examples, and answered questions if asked. Content: The intervention provided general information about domestic violence to alert health professionals (prevalence, risk factors, consequences on women’s health, pregnancy, and children) and guidelines on screening and how to deal with women disclosing domestic violence.	Only 4.1% of patients were screened for domestic violence during pregnancy followup. The 38.1% of healthcare professionals had never screened for domestic violence, only 14.3% stated they always did. The study identified the following barriers to screening: the presence of the partner, the lack of awareness of the need to screen, uncomfortable feelings, and the difficulty of identifying victims.

### Duration

Educational interventions varied in length, ranging from one hour and a half ^20^ to 14 weeks – 2 hours of course^18^. Programs that targeted health professionals were short-lasting one day or less^23^. In contrast, programs that targeted students lasted 10–14 weeks^18,22^. One study included a one-day workshop for student midwives^23^.

### Educational method and content

The educational programs included a variety of educational approaches such as group activities, role play, and analysis of case studies. Furthermore, most researchers focused on group activities, as resolving IPV cases during pregnancy requires a team effort. On the contrary, only 2 of the educational programs used the traditional method of delivering knowledge through presentations^18,20^. The programs had similar content, including raising awareness of violence, defining it, discussing gender roles, the impact of IPV on women’s health, referral agencies, and the laws regarding violence in each country. Two of the studies included survivor experiences of maternity care during pregnancy^24,25^. The effect of IPV on child health was addressed in 3 studies^20,23,25^. Experts in gender-based violence, including midwives, academics, doctors, and psychologists, designed most of the programs.

An overview of all the characteristics of educational training programs on intimate partner violence during pregnancy for midwives and student midwives is shown in Table 2.

**Table 2:** Overview of the characteristics of Educational Training Programs on intimate partner violence during pregnancy for midwives and student midwives from January 2010 to March 2023

Characteristics	Specific characteristics
**Duration**	One day (hours have not been identified) Four days (hours has not been identified) One hour and a half 7 hours 10 sessions for 2 hours
**Educational method**	Multidisciplinary sessions Lectures Theory Role-playing Practice in screening Group activities Watch videos Case reports Discussion
**Content**	IPV awareness Types of IPV Gender roles Social and gender equality Risk factors Practical training for routine Inquiry for IPV Documentation Why women may choose to stay with a violent partner. Health effects on women’s, fetal, and child Stereotypical attitudes and myths about IPV Guidelines on screening Screening tools Role and responsibilities of midwives/other health professionals in the elimination of violence Prevention law/legal aspects of violence against women Violence screening tools Referral to the advocacy service Available supportive services for IPV sufferers How to assist IPV sufferers IPV survivor experiences during pregnancy Development of security plans and notification forms. Health Child protection issues/law. Self-care strategies Participants debrief

### Previous experience with IPV training

Five studies (n=5) reported that the majority of participants in their studies had not received any other IPV-related education before the educational intervention. Furthermore, maternity professionals were significantly more likely to have received training for IPV than genitourinary medicine professionals^12^. Additionally, it has also been shown that even midwives and midwifery students had limited knowledge or experience of violence against women^18^.

### Areas of effectiveness of the reviewed educational programs on IPV

One study^11^ showed an improvement in participant midwives’ knowledge about IPV during pregnancy. Furthermore, this study concluded that following the educational program, midwives felt more confident in assessing abusive experiences, especially during antenatal visits. Another study^21^ reported that following the educational program, most participant midwives were able to identify at least one IPV survivor three months after the intervention.

Several studies^11,21,24,25^ reported that training on IPV increased participants’ knowledge, self-perceived responsibility for the identification of the victims, and self-confidence in supporting women who had experienced violence. The knowledge on screening women for abuse was increased in more than 60% of the participants, specifically in the areas of abuse disclosures, awareness of referral pathways, and how to work with multidisciplinary teams to support women who had experienced abuse^25^.

Students developed more positive attitudes toward violence and gender roles following the training^22,23^. Furthermore, their confidence levels increased in four areas: responding to and discussing domestic violence with women, assisting women in accessing support, discussing women’s experiences of violence, and accessing support for themselves after working with a woman who discloses violence. Professionals mentioned several barriers to screening pregnant women for IPV, including the presence of the partner, a lack of awareness of the need to screen, uncomfortable feelings, and difficulty identifying victims^20^. Additionally, some professionals felt that their workplace did not allow adequate time to respond to disclosures of IPV^25^.

## DISCUSSION

Overall, the scoping review highlighted the lack of educational programs for intimate partner violence in pregnancy for the professionals and students of midwifery, as most of them had not received any training^26^. Midwives often feel unprepared to work with women in this important area of midwifery practice^23^. ΙPV knowledge of the midwives and nurses was poor, as reflected by their very low percentage of correct answers of general knowledge about violence^27^. These inadequacies in knowledge, attitudes, and practices could affect the care of those who experienced IPV as a result of their negative experiences in healthcare^28^.

Additionally, some students had little sensitivity to the subject and wrong ideas about violence, and over half of students reported that the subject was not addressed in their program of study^29^. However, after the training, results show improved knowledge and promoted preparatory/reinforcing behaviors^30^. Studies suggest that health services and workplaces that incorporated training on IPV and had clear referral pathways helped midwives to routinely ask pregnant women about IPV, and they felt more confident in managing positive disclosures^31^. Furthermore, IPV education for midwives is imperative to commence at the undergraduate level and continue post-registration to ensure skills and knowledge base are maintained^16^. It has also been shown that pregnant women in antenatal settings may be more likely to disclose IPV when screened by professionals^32^.

Fortunately, there are a number of tools to diagnose IPV (in all forms: physical, sexual, and psychological) in pregnant women^33^. For example, the RADAR tool^34^ includes the following steps: 1) Routinely screen adult patients, 2) Ask direct questions, 3) Document your findings, 4) Assess patient safety, and 5) Review options and referrals. Another tool is the HITS Tool for domestic violence (Hurt, Insult, Threaten, and Scream). This self-report tool has been used in different populations and both genders and provides a sense of privacy as it does not ask for details. Finally, the HITS tool is not time-consuming as it only includes four questions using the Likert scale. Finally, the most widely used IPV screening tool in the pregnant population is the AAS Tool^34^. AAS is a five-item screening tool including the following questions: 1) ‘Have you ever been afraid of your partner or someone else?’; 2) ‘Have you ever experienced that a partner or ex-partner has done things to make you feel afraid of them?’; and 3) ‘Done things to try to intimidate you or to control your thoughts, feelings or actions? Hit, kicked, pulled you by your hair or otherwise physically hurt you? Forced you to have sexual activities against your will?’. Finally the AAS tool has been tested in obstetrics–gynaecology outpatient practices and among different ethnicities^31^.

More recently, during the lockdown periods because of COVID-19, there was a pilot study in a large US healthcare establishment that utilized an application (i.e. MyHealthyPregnancy app) to monitor risks during pregnancy, including IPV. Through this application, patients can be screened for IPV remotely. For example, users received in-app messages that stated: ‘Are you concerned about your safety? Take the pregnancy safety quiz’, etc. The study showed a slight increase in IPV during lockdowns^35^.

This scoping review confirms that midwives prefer to develop a relationship with women before asking about IPV^36^. This finding emphasizes the need for an organizational change in the healthcare setting to adequately respond to IPV^27^, with continuity of education and midwifery care being the key. As long as health professionals keep being quiet about violence, the victims will carry on avoiding sharing their experiences and worries.

### Strengths and limitations

A notable advantage of this review is that most items outlined were conducted separately by two assessors. The strengths of this study are based on the satisfactory number of primary studies included in the review covering a wide range of implementation years.

Several limitations to this review study need to be acknowledged. During the literature search, only studies containing the keyword midwives were included. However, due to the limited number of studies involving registered midwives, studies involving student midwives had to be included to broaden the scope of this review. Studies including health professionals who were not identified as midwives were excluded.

Another limitation of this study is the unavoidable introduction of language bias due to the use of articles in the English language only. Furthermore, the studies included in this review described educational programs in Asia, Europe, and Australia. The absence of studies from America and Africa is noted. It is, therefore, recognized that programs in many parts of the world that are given in different languages may exist but remain unpublished. However, further in-depth research will be required to ensure global knowledge about published or unpublished programs for registered midwives and midwifery students.

## CONCLUSIONS

The educational programs for intimate partner violence can be beneficial for professional midwives and students despite their lack. These programs could impart knowledge and skills to participants to identify incidents of intimate partner violence, address them, and protect abused pregnant women. There is an urgent need to create new programs that must adapt to new clinical practices and recommendations for midwifery education on intimate partner violence during pregnancy. These new programs should be integrated into midwifery university programs, as well as in continuing education programs for midwives who work in hospitals, the community, or are independent. Recognizing the problem can be the best way to prevent it. Future research will concentrate on utilizing the results of this research to create a new educational program and to investigate the effectiveness of knowledge and preparedness of midwives to conduct routine inquiries about intimate partner violence with women during pregnancy.

## Supplementary Material



## Data Availability

The data supporting this research are available from the authors on reasonable request.
